# Optimising impact and sustainability: a qualitative process evaluation of a complex intervention targeted at compassionate care

**DOI:** 10.1136/bmjqs-2017-006702

**Published:** 2017-09-15

**Authors:** Jackie Bridges, Carl May, Alison Fuller, Peter Griffiths, Wendy Wigley, Lisa Gould, Hannah Barker, Paula Libberton

**Affiliations:** 1 Faculty of Health Sciences, University of Southampton, Southampton, UK; 2 NIHR CLAHRC Wessex, Southampton, UK; 3 Institute of Education, University College London, London, UK

**Keywords:** health professions education, humaneness, implementation science, organizational theory, teamwork

## Abstract

**Background:**

Despite concerns about the degree of compassion in contemporary healthcare, there is a dearth of evidence for health service managers about how to promote compassionate healthcare. This paper reports on the implementation of the Creating Learning Environments for Compassionate Care (CLECC) intervention by four hospital ward nursing teams. CLECC is a workplace educational intervention focused on developing sustainable leadership and work-team practices designed to support team relational capacity and compassionate care delivery.

**Objectives:**

To identify and explain the extent to which CLECC was implemented into existing work practices by nursing staff, and to inform conclusions about how such interventions can be optimised to support compassionate care in acute settings.

**Methods:**

Process evaluation guided by normalisation process theory. Data gathered included staff interviews (n=47), observations (n=7 over 26 hours) and ward manager questionnaires on staffing (n=4).

**Results:**

Frontline staff were keen to participate in CLECC, were able to implement many of the planned activities and valued the benefits to their well-being and to patient care. Nonetheless, factors outside of the direct influence of the ward teams mediated the impact and sustainability of the intervention. These factors included an organisational culture focused on tasks and targets that constrained opportunities for staff mutual support and learning.

**Conclusions:**

Relational work in caregiving organisations depends on individual caregiver agency and on whether or not this work is adequately supported by resources, norms and relationships located in the wider system. High cognitive participation in compassionate nursing care interventions such as CLECC by senior nurse managers is likely to result in improved impact and sustainability.

Despite renewed focus on compassion in UK healthcare and internationally, there is a lack of agreement on how best to promote and sustain compassionate healthcare.^1 2^ There is an understandable temptation to deploy solutions solely aimed at caregivers (such as medical or nursing staff) seen as lacking the relational capacity to engage in caring relationships with patients. There is however no compelling evidence that such initiatives are effective in improving individual practice or patient outcomes.^3 4^ Individual capacity for relational practice depends on individual agency and on features of workplace context, including resources (such as time to perform relational work), professional and/or institutional norms about what constitutes legitimate work, and relationships that shape, for instance, the extent of social support afforded to caregivers for their relational work.^5–10^ Interventions targeted at how workplace conditions may better foster individual caregiver relational capacity may therefore be more effective than those solely focusing on improving individual caregivers’ personal attributes.

Recent years have seen the development and evaluation of a number of interventions focused on improving compassionate care at team rather than individual practitioner level.^11–16^ Such interventions have typically been facilitated by a senior nurse, using reflective learning, action research and/or appreciative enquiry to work with ward-based nursing staff (often using patient stories and/or observations of practice) to strengthen support for existing good practice and make changes where needed. The evaluative focus of these studies is the mechanisms for change used, particularly the processes deployed to shape the practice changes made. They also often include an analysis of the enablers and barriers to change. However, they do not examine in depth the process of implementation itself and so fail to systematically identify the contexts in which successful implementation is more likely or, where contexts are not receptive, how resources, relationships and norms in the wider system may need purposeful restructuring in order to support implementation and sustain longer term change.^17 18^ The analysis presented in this paper draws on normalisation process theory (NPT) to more thoroughly investigate the process of implementing an intervention aimed at supporting the delivery of compassionate care by hospital teams.

Creating Learning Environments for Compassionate Care (CLECC) is a team-based workplace educational programme focused on creating a sustainable ‘expansive’ learning environment, through leadership and team practices (dialogue, reflective learning, mutual support, role modelling), that enhances team capacity to provide compassionate care (figure 1).^19–22^ The team focus draws on research indicating associations between work group mechanisms that promote shared norms, social support for individual members and care quality.^8 9 23 24^ CLECC is designed to optimise and sustain personal and team relational capacity, that is, capacity to embed and sustain relational approaches in practice within a complex and dynamic organisational context.

**Figure 1:**
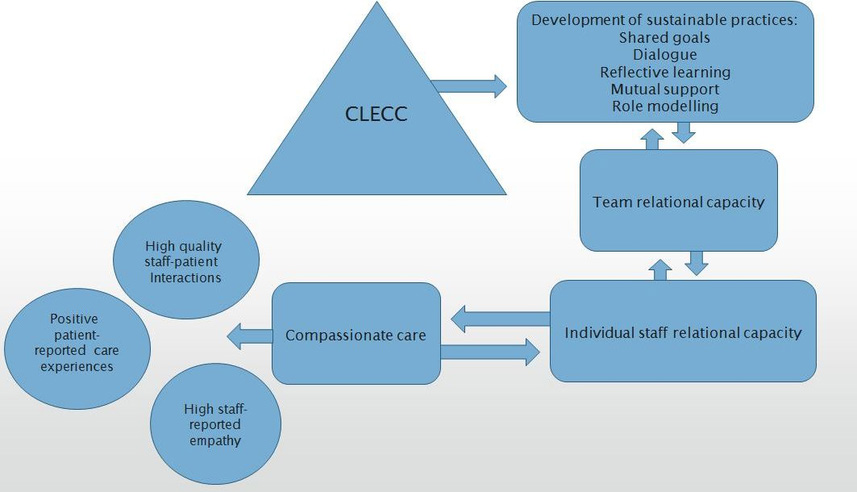
Overview of Creating Learning Environments for Compassionate Care (CLECC) programme theory.

CLECC’s 4-month implementation period, facilitated by a senior practice development nurse (PDN), includes a combination of set activities that represent expansive workplace practices, with a view to embedding these practices to support sustainability (table 1): regular CLECC meetings between ward manager and matron; ward manager action learning sets, including one focused on influencing senior managers; team learning activities, including climate analysis and values clarification; peer observations of practice; team study days; mid-shift 5 min cluster discussions; and twice weekly reflective discussions.^19 25^ Teams also develop a learning plan to be shared with a senior hospital manager that includes sustainability measures for practices that underpin the delivery of compassionate care.

**Table 1:** Creating Learning Environments for Compassionate Care (CLECC) set activities

Activity	Month 1	Month 2	Month 3	Month 4
Ward manager action learning sets	Session 1/setting up set, setting ground rules	Session 2/workplace climate/team values/valuing staff	Session 3/enhancing team capacity for compassionate care	Session 4/influencing senior managers
Team learning plan	Introduce and discuss	Discussion and draft by ward leader	Finalise, identify resources needed to support, present	Senior manager feeds back response to team plan
Ward manager/matron meetings	Introduce and discuss	Ongoing – every 2 weeks	Ongoing – every 2 weeks	Ongoing – every 2 weeks
Peer observations of practice	Identify two volunteer observers from staff team	Train observers	Observations of practice	Feedback observations of practice
Team study day (all staff)	Team analysis of workplace climate/values clarification	–	–	–
Mid-shift cluster discussions (all staff)	Ongoing	Ongoing	Ongoing	Ongoing
Reflective discussions (twice weekly) (all staff)	‘I feel valued at work when…’ exercise	Team values clarification exercise; Best Practice for Older People (BPOP)^25^ activities	BPOP activities^25^; team learning+service user feedback plan discussions	Reflections on feedback from observations of practice

## Methods

This qualitative process evaluation used NPT to identify and explain the extent to which CLECC was implemented into existing work practices and to identify how CLECC can be optimised to support sustained compassionate care delivery in acute settings. NPT focuses on four dynamic processes (coherence, cognitive participation, collective action and reflexive monitoring) that motivate and shape implementation processes. Part of a wider feasibility study of CLECC,^26^ this evaluation focused on:

1. exploring how and in what ways the new practice was initially received, how individually and collectively people practically conceptualise and make sense of it (coherence)
2. assessing the degree of ownership of and participation in the new practice by key individuals and teams (cognitive participation)
3. identifying the work that individuals and teams do to enact the new practice (collective action)
4. exploring the perceived impact of the new practice on staff work and on patient outcomes (reflexive monitoring).

The CLECC intervention was introduced to four inpatient wards in two general hospitals in England in 2015. Wards with high proportions of older patients in which the ward manager was expected to remain in post for at least 6 months were recruited through ward manager agreement. Wards specialised in either medicine for older people (n=3) or trauma and orthopaedics (n=1). Data were gathered between May 2015 and May 2016.

Individual face-to-face semistructured interviews were undertaken with staff over a 12-month period beginning at the outset of the implementation period followed by two further interview rounds (at 3–6 months and 7–12 months). We purposively sampled volunteers to capture variations in staff grade and ward. Recruits were invited to further interviews so that variations over time could be tracked. Where there was attrition, new individuals were recruited to ensure ward and grade variation was maintained. Senior nursing managers were invited to one interview in the final phase. Interview schedules reflected NPT dynamic processes and changed over time to reflect implementation stage. Interviews were conducted by university researchers and lasted on average 46 min. Interviews were audiorecorded, transcribed verbatim and transcripts checked for accuracy. Each PDN, seconded to the role from existing NHS employment, kept detailed field notes. A sample of CLECC learning activities was also observed by university researchers (n=7 over 26 hours). Staffing data were gathered through a ward manager questionnaire.

Data were first analysed using systematic reading, familiarisation and open coding, undertaken independently by research team members and then in collaborative data analysis workshops. A preliminary coding frame focused on implementation and mechanisms of impact. All interview data were coded against this frame, the use of constant comparative methods enabling the generation of new categories and the comparison of data in relation to these categories. Narrative data summaries and matrix/charting techniques were then used to facilitate comparison with the NPT framework to test and refine emerging theories of implementation processes. All data from observer and PDN field notes, and from quantitative analyses of staffing data, were then systematically interrogated and compared against these emerging theories, the purpose being to use multiple perspectives to elicit more complex and situated understandings.^27 28^

Ethical approval for the study was granted by the Social Care Research Ethics Committee 14/IEC08/1018.

## Results

In total, 47 interviews were conducted with ward managers (n=4 people), deputy ward managers (n=2), staff nurses (n=8), healthcare assistants (HCAs) (n=7), senior hospital nurses (n=2) and PDNs (n=2). Thirteen people were interviewed once, two people twice and ten people three times. Eleven people declined further interviews after one interview and two people after two interviews. Ward-based interviewees had worked on their current ward between 2 weeks and 14 years, on average 4 years. Two study days and five action-learning sets were observed in full.

Findings illustrate the work that staff needed to do, individually and collectively, to implement CLECC in practice. While many of the individual elements of CLECC were possible to implement during the implementation period, sustaining this work beyond this time was difficult for some ward teams to achieve. The findings that follow explain why this was the case.

### Coherence: CLECC as limited set of concrete practices versus underpinning philosophy

Interview and observation data clearly indicated that all care staff were able to articulate activities associated with the CLECC intervention. Staff valued the principles behind many concrete CLECC activities, appreciating the focus on staff well-being and consequent impact on patient care quality. For registered nurses (RNs), the CLECC principles resonated with their aspirations for successful team working and patient care. For HCAs, this was a new and welcomed way of thinking about their workplace. Beyond the activities staff were directly involved in, they struggled to visualise the purpose and potential of CLECC. Staff tended to associate CLECC with cluster discussions that took place part way through each shift, thus providing an opportunity to gather as a team and check on each other’s well-being.

*“So, whereas before they might know that orange bay is heavier than green bay, they might not necessarily have volunteered to go and help. Now they are much more aware that if they are going – well actually we’re struggling – well, we’re not, we’ll come and help you and I think that’s because of the [clusters] and the fact that we’re all sitting down and going – is there anything we can do to help you? And if they are going – well actually I’ve got a really poorly patient, so I’ve been struggling with the others – right – well then – we’ll come and help you. And it’s made them more aware of each other.”* N003^[i]^ (HCA)

All staff attended the study days and, on prompting, were able to link these sessions with CLECC. Participating in a study day where only other team members were present was considered unusual and was welcomed. Staff saw the study day as a way of ensuring that they were working together and an opportunity to engage with the ward vision, which was not previously explicit. The most important aspect of the study day was the chance to get to know each other, which staff reported they had not had the opportunity to do previously.

*“We had the study days and they were all very good and I found that I got to know the different people within those study days, or how they felt and I thought – oh, I didn’t know that. So that was useful.”* N001 (Staff Nurse)

The ward managers and PDNs charged with facilitating CLECC were involved in a wider range of CLECC activities; these individuals and the senior nurse managers were clearly able to articulate the underlying philosophy of CLECC and to identify associated behaviours.

*“To me CLECC is about giving staff tools to ensure that they support themselves to do a hard job. So it’s about providing – a nurse with the knowledge of what they need to deliver… compassionate care or high-quality person-centred care, whatever you want to describe it as – every day, at a high quality standard, is what we have to aim for, but also with you having some insight into how your behaviour affects both your patient and your staff.”* SN002 (Director)

On average over a third of staff left over the course of the study, consistent with other wards we were monitoring, and one senior manager viewed staff turnover as a result of CLECC as a positive outcome:

*“[CLECC] exposed some practices, provided a culture where people could talk openly about how other members of staff made them feel; there has been a bit of a churn, so maybe some people that needed to go. People have now felt they’ve got a voice and, again, if people aren’t doing what we need them to do, then they need to go.”* SN002 (Director)

But there was no provision for inducting newly arrived staff into CLECC, limiting their opportunity to make sense of CLECC.

In summary, although ward staff appreciated the potential value of CLECC, their understanding of CLECC was limited to and shaped by the concrete activities that they experienced. Additional knowledge about the underpinning principles of CLECC did not filter down to the team as a whole, with no evidence that coherence improved over time from the original induction into CLECC activities.

### Cognitive participation: staff keen to participate but not sure who should drive it forward

Staff were keen to participate in CLECC, but it was not always clear whose responsibility it was to ensure it happened. Each PDN worked simultaneously with two wards in their allocated hospital, organising specified CLECC activities. Each took a different approach and this influenced the degree of ownership of the intervention by the ward staff. One PDN had an autocratic style of leadership and deliberately undertook to transfer ownership for making things happen, seeing her role as informing teams how things should be done by them:

*“[The PDN] was very adamant that it wasn’t her responsibility to do cluster, it could have been anybody’s….”* N008 (HCA)

After the implementation period, staff on those wards reported that cluster discussions were no longer taking place because no one senior was making them happen. The other PDN had a more democratic style of working and actively worked with staff to make CLECC more flexible and fit better with resource pressures.

*“It [cluster meeting] doesn’t always stick to that time. It kind of depends how it’s going. So we’ve had like busy days when stuff’s been happening on the ward. At one point they [nursing staff] kind of ask permission to make it [cluster meeting] later, it’s kind of sad. But I’m like… ‘yeah, do it whatever time it works in the ward. If we can do it, that’s a bonus’. So quite often it’s the [HCAs] asking for it [cluster meeting].”* N035 (PDN)

Although this PDN and more senior team members initially originated the cluster discussions, as the intervention became embedded over time others, including HCAs, called them without waiting for more senior initiation. Cluster discussions on these wards continued to run after the implementation period.

*“They [HCAs] will remind whoever is in charge of the ward, and say ‘Are we having a [cluster] today?’ I’ve seen that quite a few times.”* N035 (PDN)

CLECC also gave staff, including HCAs, the opportunity to see themselves as innovators, providing a mechanism through which individuals could articulate their ideas for improving practice on the ward. Staff felt more empowered than before to respond to ideas and to implement change, approaching the ward manager and the matron simultaneously, when previously communication was directed through the ward manager.

*“Quite a few of the staff have got involved in various different things that have come out of the study days, what they wanted to change, and thought they could do better. And they’ve gone off and sort of little groups, or twos and threes, and are bringing that stuff back, passing it through the Matron.”* N030 (Ward Manager)

Not all ideas were implemented in practice, which appeared to be linked with uncertainty about whose role it was to realise them or to authorise them. Staff clearly had an expectation that the ward manager or matron had the requisite authority to realise the ideas and the lack of ‘follow through’ was demoralising for the staff involved.

*“Some of them felt a little bit disappointed that they’d made these suggestions and took their time to do them and then no one really followed it through or said – yes, we can use that or no we can’t. It just got left.”* N001 (Staff Nurse)

Everyone interviewed reflected that they saw CLECC as a way to build the team and improve care, and this ethos underpinned their participation in prescribed activities. Consequently study days and action learning sets were all well attended. Fortnightly CLECC meetings between ward managers and their matrons did not go ahead in one hospital site, indicating a lack of clarity about the role of the matron in making CLECC happen.

*“Both ward [managers] felt that there has been a negative impact from the lack of support from the matron. Items identified by the nursing teams that were considered areas requiring improvement were unsupported and even in some instances rejected.”* N036 (PDN field note)

Already established meetings between ward managers and their matron at the other hospital site appeared to be linked with a more proactive matron role in supporting CLECC.

*“So my matron’s been very supportive the whole way through; we’ve kept in regular contact. She’s been asking for updates, she’s known about the interventions that we’ve done on the ward and has been really supportive.”* N034 (Ward Manager)

Consequently, while the majority of staff were keen to participate, the extent to which individuals saw it as their role to make CLECC happen varied between professional position and level in the organisation.

### Collective action: participation shaped by organisational context

Whether or not the activities went ahead as planned was mediated by the extent to which the proposed activity harmonised with the priorities of the wider hospital organisation and resources available to the ward team. A particular influence was the organisational priority afforded to material patient care activities over CLECC activities in the context of high patient care workloads. Staff reported struggling to find the time to engage with cluster discussions. The planned 20 min reflective learning sessions proved impossible to integrate into ward practice. CLECC’s flexibility enabled staff to develop strategies that partly overcame these barriers.

*“Because [the clusters are] 5 min you can work it and actually if you’re having a day where you’re too busy to run them, then that’s the day that you realise that you need to go round and make sure everyone’s okay…And I think that’s definitely been my biggest struggle throughout it all – it’s just being able to release staff to do things.”* N005 (Ward Manager)

Staff reported that senior hospital managers had endorsed the work that had resulted from the CLECC intervention, suggesting that the benefits were visible and valued outside of the immediate ward team.

*“They seemed to be really positive about it and [visiting senior manager] said – ‘if this is working for you, continue.’” *N009 (HCA)

Nonetheless, staff’s participation in CLECC activities was viewed as secondary importance to providing patient care. The degree of support from individual matrons also seemed linked to leadership resources being made available by the organisation to support the team with CLECC:

*“I assumed that my matron was working with the ward [managers] on a weekly basis but I doubt it was what I expected it to be. So we should have put more nursing leadership resources into it, just to provide that support and recognise it.”* SN002 (Director)

Many cluster discussions proved possible to integrate into the working day and went ahead during the implementation period but were less readily convened when patient care demands were very high and staffing resource was low. CLECC properties of plasticity enabled staff to develop and adapt practices that suited local circumstances but were constrained by the available resources and priorities of the wider organisation.

### Reflexive monitoring: valued by staff but challenging to sustain

Staff reported benefits to personal well-being and capacity to care from CLECC participation. They spoke of engaging more consciously and deliberately with patients as people, prioritising this over the completion of tasks. Staff reported that their practice was already compassionate, but CLECC had given them opportunities to value these practices and to make further commitment to compassion.

*“CLECC, for me, is about giving the staff the empowerment to feel like they can sit and do things with patients that are compassionate rather than task orientated, so rather than just doing the [observations] and just doing the washes, just having a chat with the patient about their life, their family or sitting and doing an activity with them; rather than just, we’ve got to get the washes done, we’ve got to get the observations done – which do still need to be done but it’s about giving the staff that that empowerment of being able to say, let’s do something a bit different.”* N034 (Ward Manager)

CLECC was associated with an improvement in staff morale and staff well-being and viewed as impacting positively on patient care. Interestingly, some of the legitimacy for CLECC practices seemed to come from the fact that they were part of the CLECC intervention and perhaps a research study. One interviewee cited an instance in which a senior manager visiting the ward came across a cluster discussion, which were also used by some teams to make sure that staff had a drink of water.

*“I don’t know who it was, but someone very high in the hospital [came to the ward] and was like, ‘why are people standing and drinking on the corridor?’”* N025 (HCA)

Once the manager was told the cluster discussion was part of CLECC, she was reported to have then understood the purpose behind an activity considered to be unusual enough to be remarked on.

The improved team working reduced the burden for some staff and provided opportunities to undertake activities that previously would have been rare occurrences.

*“But because of the task orientated work – we’ve managed to go, right, we’ve finished, [they] haven’t and then so we can go, right, we’ll give you guys a hand and then we can all be finished together. And then that means we’ve got more time to do things that we might not be able to normally do, like – wash someone’s hair, do their nails.”* N009 (HCA)

The principles that underpin CLECC appeared to be well embedded into the teams, even several months after the end of the implementation period. Some of the wards continued with the cluster discussions, and even on wards where they had not continued, staff valued them as an indicator of supporting each other and working together. Attention to supporting each other appeared to have increased the relational capacity of individual team members and the team as a whole.

## Discussion

This study aimed to identify and explain the extent to which the planned CLECC intervention was implemented by nursing staff into existing work practices, to enable conclusions to be drawn about how interventions of this kind can be optimised and sustained to support compassionate care in acute settings.

While CLECC had limited coherence for some staff, it was welcomed by teams and served as a broader stimulus to collective action. CLECC developed cultures in which reflection, learning, mutual support and innovation were legitimised within the work-team, and in which expertise was seen to be distributed more widely between managers, RNs and HCAs. CLECC moved all the teams further along the continuum to becoming more expansive learning environments,^20^ but implementation was mediated for all by the context of working in an acute hospital environment. Staff highlighting what they valued about CLECC illuminated the stark realities of team-working in such settings. The struggle to find the time to participate in CLECC reflects the pressure on staff to be constantly engaged in material patient care activities. Staff valued the cluster discussions because, otherwise, there was little opportunity to support each other’s well-being. They appreciated the study days because they could get to know each other as people. They valued CLECC because otherwise they were lone workers, sharing working time and space with other team members, but not actually working as a team at all. The intensification of nursing work due to rising patient complexity, in parallel with the application of increasingly stringent financial efficiency quotas, is well documented.^9 29 30^ These findings paint a rich picture of the consequences for staff experiences at work and explain associations between hospital work-team climate and staff well-being reported elsewhere.^23 24^

Staff at all levels were able to identify the benefits to patient care of ward staff engaging in CLECC activities, echoing other findings that the creation of unmanaged spaces for work-team members to ‘take shelter’ provides the potential for valued learning and social support for difficult work with clients.^8 9^ The findings confirm that intervening at work-team level can be successful, confirming an association conjectured from other research.^19 23 24 31^ In spite of high workloads, CLECC empowered staff to reflect on local norms governing team practice, and on the relationships and resources that aligned with them, and to make some changes, indicating that collective agency can play a part in shaping relational capacity at individual and work-team level.^9 32^ However, we also found that implementation was uneven between teams, particularly over the longer term, reinforcing the value of paying attention to the sustainability of complex interventions beyond initial set-up.^33–35^

Factors outside of the direct influence of the ward teams mediated the impact and sustainability of the intervention, in particular the institutional norms that legitimated staff’s participation (or not) in CLECC activities and the interpretation by more senior figures (including PDNs, matrons and senior hospital managers) of what CLECC was and their role in supporting it. While CLECC draws on principles of democratic working, and we saw how HCAs in particular were enabled to take a lead in some CLECC activities, its longer term success relied on cognitive participation from more senior members of the hierarchy. The authority that ward staff had to control how they spent their time, to innovate and to afford their own and colleagues’ well-being some priority varied between teams and was dependent on the signals, or ‘invitational qualities’^32^ from these more senior figures as to what was legitimate or not. These findings that nurses do not control the conditions in which they work echo extensive research on the curtailment of professional autonomy in publicly funded healthcare, and the particular position that nursing as a profession occupies.^5–7 30 36–38^ Matrons are the point at which organisational drivers, often business imperatives, must align with professional imperatives and the needs of frontline teams and their patients. The hybrid role and competing identities for nursing managers of this kind have been highlighted elsewhere,^30 39 40^ and it is unsurprising that we identified different approaches to managing this key role. While the current CLECC activities related to senior manager participation appear to have aided coherence, findings suggest that additional activities targeted at improving their cognitive participation may be needed.

Our findings indicate that higher and more sustained impact for interventions such as CLECC may only be possible through more substantial restructuring that reshapes the conditions in which people are able to act.^17 18^ We support Parker’s assertion that caregiving organisations need to be designed to enable caregivers to access functional work-teams within which they can interpret their experiences, and we have identified a number of concrete but modifiable barriers that merit attention in such design, including lack of time and institutional rules that undermine the value of staff-to-staff social support.^8^ They also include more clearly defining the role of nursing managers in signalling the legitimacy of staff providing each other with emotional support, supporting nursing teams to meet and learn together. Future versions of the CLECC intervention will include new activities to engage nursing managers in the implementation period, involve them in the learning activities and create opportunities for them to engage and reflect with frontline staff.

These findings reflect that relational work in caregiving organisations depends on individual caregiver agency and on whether or not this work is adequately supported by features of the wider system. Relational capacity may thus be regarded, not just as a property of individual practitioners, but a modifiable and situated property of work-teams. The limitation that institutional norms of legitimate nursing work placed on staff finding time to meet together raises the prospect of lack of relational capacity at a wider system level, and suggests that wider restructuring beyond middle manager roles may in fact be needed to effect substantial and sustained change.

## Footnotes

i N denotes nursing team membership, SN denotes senior nursing manager, number represents order of entry into the study.
